# The cotton charcoal rot causal agent, *Macrophomina phaseolina*, biological and chemical control

**DOI:** 10.3389/fpls.2023.1272335

**Published:** 2023-09-19

**Authors:** Ofir Degani, Asaf Gordani, Elhanan Dimant, Assaf Chen, Onn Rabinovitz

**Affiliations:** ^1^ Plants Sciences Department, Migal—Galilee Research Institute, Kiryat Shmona, Israel; ^2^ Faculty of Sciences, Tel-Hai College, Upper Galilee, Tel-Hai, Israel; ^3^ Soil, Water and Environment Department, Migal—Galilee Research Institute, Kiryat Shmona, Israel

**Keywords:** azoxystrobin, crop protection, field study, fungus, integrated control, irrigation, *Trichoderma*, real-time PCR

## Abstract

The fungus *Macrophomina phaseolina* causes charcoal rot disease (CRD) in cotton, whose symptoms develop in the late stages of growth and result in wilting and death. Despite significant research efforts to reduce disease incidences, effective control strategies against *M. phaseolina* are an ongoing scientific effort. Today’s CRD control tends toward green options to reduce the chemicals’ environmental footprint and health risks. Here, different *Trichoderma* species were examined separately and in combination with Azoxystrobin (AS) in semi-field open-enclosure pots and a commercial field throughout a full season. In the pot experiment, the *T. asperellum* (P1) excelled and led to improvement in growth (13%–14%, day 69) and crops (the number of capsules by 36% and their weight by 78%, day 173). The chemical treatment alone at a low dose had no significant impact. Still, adding AS improved the effect of *T. longibrachiatum* (*T7507*) and impaired P1 efficiency. Real-time PCR monitoring of the pathogen DNA in the plants’ roots at the harvest (day 176), revealed the efficiency of the combined treatments: *T. longibrachiatum* (T7407 and T7507) + AS. In a commercial field, seed dressing with a mixture of *Trichoderma* species (mix of P1, T7407, and *Trichoderma* sp. O.Y. 7107 isolate) and irrigation of their secreted metabolites during seeding resulted in the highest yields compared with the control. Applying only AS irrigation at a low dose (2,000 cc/ha), with the sowing, was the second best in promoting crops. The molecular *M. phaseolina* detection showed that the AS at a high dose (4,000 cc/ha) and the biological mix treatments were the most effective. Reducing the AS chemical treatment dosages by half impaired its effectiveness. Irrigation timing, also studied here, is proven vital. Early water opening during the late spring suppresses the disease outburst and damages. The results demonstrated the benefits of CRD bio-shielding and encouraged to explore the potential of a combined bio-chemo pest control approach. Such interphase can be environmentally friendly (reducing chemical substances), stabilize the biological treatment in changing environmental conditions, achieve high efficiency even in severe CRD cases, and reduce the development of fungicide resistance.

## 1 Introduction

The fungus *Macrophomina phaseolina* is distributed in the soil and can attack more than 500 plant species from about 75 families (Marquez et al., 2021). Disease severity is correlated with viable sclerotia in the soil, the pathogenic variations of the fungus (Iqbal and Mukhtar, 2014), and host specialization (Su et al., 2001). The pathogen causes charcoal rot disease (CRD) in cotton (*Gossypium* spp. L.), with symptoms that develop mainly in the late stages of growth. These include drying leaves and stems, plant wilting, and death (Marquez et al., 2021). In recent years, the disease has increased in cotton fields in Israel, possibly due to the transition to susceptible extra-long staple (ELS) Pima-type (*Gossypium barbadense*) variety and the climatic conditions changes (Cohen et al., 2022). It is now widely agreed upon that CRD significantly impacts Israeli cotton agriculture.

The CRD fungus, *M. phaseolina*, can generate microsclerotia or pycnidia within the host plant’s roots and stems, allowing it to persist in crop residue and soil over an extended period (Romero Luna et al., 2017). The pathogen secretes toxins and cell wall–degrading enzymes (Islam et al., 2012), enabling it to penetrate and kill the host plant cells. Plants with densely packed, sturdy lower stems, like cotton, are susceptible to the harmful effects of toxins, which can cause wilting without any apparent early symptoms (Cohen et al., 2022). Indeed, during the cold, early stage of the growing season, signs of CRD may not be noticeable. However, symptoms manifest as the weather gets warmer, and the crop develops through flowering and fruit set. These symptoms can worsen if the plants are experiencing stress due to heat or drought (Romero Luna et al., 2017). The symptoms’ enhancement is because *M. phaseolina* thrives under hot (ca. 30 °C and above) and dry conditions, commonly found in countries with a Mediterranean climate, such as Israel (Cohen et al., 2022). As the global climate warms up, CRD will likely cause more crop losses and spread to new areas.

Despite significant research efforts to reduce disease incidence, with varying degrees of success, developing effective control strategies against *M. phaseolina* remains an ongoing scientific effort (Iqbal and Mukhtar, 2020b; Marquez et al., 2021). Reducing environmental stressors that affect plants is one aspect that should be applied alongside other approaches, as *M. phaseolina* took advantage of non-optimal growing conditions (Manici et al., 1995; (Zveibil et al., 2012; Cohen et al., 2022). The development and quality of cotton plants and the prevalence and severity of CRD in cotton fields can be influenced by the amount and timing of irrigation, making them crucial factors for consideration (Cohen et al., 2022).

Crop rotations are ineffective because the fungus has a high saprophytic capacity (Almeida et al., 2001). The wide range of hosts and the long survivability of the fungus in the soil make controlling the disease particularly challenging (Marquez et al., 2021). Host resistance is the most sustainable management method, which requires identifying resistant germplasm (Aly et al., 2006). However, the complexity of the genetic control of charcoal rot resistance makes it difficult to integrate the resistance into leading cultivars, posing a significant challenge.

Without commercially preferred resistant hybrids, systemic fungicides are almost the only approach to reducing CRD damage (Bashir et al., 2017). According to Iqbal and Mukhtar (Iqbal and Mukhtar, 2020b) and Marquez et al. (Marquez et al., 2021), there is an urgent need for effective fungicides against this pathogen. In Israel, using fungicides reduces *M. phaseolina* occurrence and disease intensity in melons. However, this treatment was inefficient in cotton (Cohen et al., 2012). Still, various fungicides, such as Carbendazim, Difenoconazole, Benomyl, and Azoxystrobin (AS), have been tested against the pathogen and show some control potential. One study indicated Carbendazim and Thiophanate methyl as effective treatments (Bashir et al., 2017). Cohen et al. (2022) evaluated chemical control effectiveness in enhancing plant response to *M. phaseolina* in Israel (Cohen et al., 2022). Applying AS or boscalid, 26.7% + pyraclostrobin, 6.7% mixture as a seed coating effectively prevented seedlings’ pathogen penetration for only 12 days. Beyond this period, the roots spread out of the protected area, exposing them to pathogen penetration (Cohen et al., 2022). In another experiment, disease progress was assessed 1, 2, and 3 months after seeding following three fungicide applications. AS treatment resulted in a 15% disease incidence, whereas the non-treated control and treatments with prochloraz and prothioconazole had a disease incidence of approximately 30% (Cohen et al., 2022).

Research efforts are now directed toward biological control methods. Environmentally friendly biological agents are replacing chemical compounds due to the emergence of fungicide-resistant fungal strains and public concern about their health and environmental effects. Among these, fungi of the genus *Trichoderma* have a high pest control potential and the ability to promote growth in many crops (Hewedy et al., 2020; Iqbal and Mukhtar, 2020a; Degani and Dor, 2021; Degani and Gordani, 2022; De Oliveira et al., 2022). They are effective against many fungal pathogens of plants that develop in the soil, including *M. phaseolina* (Aly et al., 2007; Bastakoti et al., 2017; Marquez et al., 2021). These species have developed various mechanisms to disrupt pathogens, including antibiotic production, nutrient competition, and mycoparasitism. In addition, certain species can positively affect plant health and improve systemic resistance to biotic and environmental stresses (Martinez-Medina et al., 2016). Still, it should be emphasized that the lack of commercialized biological control agents and the associated problems in their use (the *Trichoderma* spp. instability in the changing environment) and management (less available production facilities and supply chain) make their practical application challenging.

In previous work, we screened eight *Trichoderma* isolates against the *M. phaseolina* pathogen, with *T. asperellum* (P1) and *T. longibrachiatum* (T7407 and T7507) selected as pest control treatments for seedlings in pots (Degani et al., 2023). The T7407 treatment significantly improved plant survival (34%), wet weight (45%), plant height (32%), and phenological development (56%) after 42 days compared with the control while reducing the pathogen root infection to zero levels (Degani et al., 2023). The results obtained in sprouts indicate the feasibility of continuing research to establish this approach in field conditions for an entire growing season, especially because the disease typically outbursts toward the season’s end.

*Trichoderma*-based biological control is a well-researched method for ecological protection; however, its practical application in farming is often hindered by natural stresses, which can result in unforeseeable control outcomes (Howell, 2003; Legrand et al., 2017; Malik et al., 2018). A new control strategy has been proposed to maintain the high effectiveness level of chemicals while significantly reducing their dosages (Ons et al., 2020). Combining different species of biological control agents with chemical pesticides at low doses throughout an entire growing season may have several advantages (Karthikeyn, 2022). These include reducing resistance development, maintaining environmental friendliness, maintaining the bio-shielding treatment stability in the environment’s unstable conditions, and achieving high efficiency, which is essential in severe disease cases (Degani et al., 2023).

Earlier reports have indicated that the combined use of low-toxic chemical fungicides and biocontrol agents can enhance the stability and effectiveness of biocontrol against plant diseases, which can lead to a decrease in the reliance on chemical fungicides. For instance, *Trichoderma harzianum* (strain SH2303) and Difenoconazole-propiconazole have been successfully applied together to control *Cochliobolus heterostrophus* southern corn leaf blight disease in maize (Wang et al., 2019). Our recent study of integrated (bio-chemical) pest control against *Magnaporthiopsis maydis* in corn (Gordani et al., 2023) proved the potential of this strategy in assisting in creating a green pest control strategy to reduce morbidity and increase crop quantity and quality.

This integrated disease control interphase has been tested here for the first time against the cause of CRD in cotton. To this end, we performed a semi-field full-season pot trial that tested the combined bio-chemo approach compared to the bio-shielding or AS treatments alone. In addition, we tested *Trichoderma*-based applications on a large commercial field scale compared with the conventional chemical treatment. The evaluations of cotton growth, yield, and disease symptoms were accompanied by quantitative real-time PCR tracking of *M. phaseolina*’s DNA inside the host roots. The field experiment was also studied using remote sensing–RGB (red, green, and blue) and thermal aerial imaging.

## 2 Materials and methods

### 2.1 Rationale of the study design

The current work tested chemical and biological control methods (separately or combined) to reduce cotton CRD. We used the already field-validated AS compound (Cohen et al., 2022) and the *Trichoderma* species that had gained success in our previous study in plate confront and seedlings pathogenicity tests (Degani et al., 2023). We previously verified that the *Trichoderma* species have no inhibition impact on seed germination and the sprouts’ initial development (Degani et al., 2023). We also tested *Trichoderma* species’ sensitivity to AS and proved that they are tolerant even to high compound concentrations (Gordani et al., 2023). The growth experiments were conducted in two steps. (1) The biological and chemical shielding was tested and compared with an integrated chem-bio control method in a semi-field (open-enclosure) full-season pot trial. This step lets us carefully control the soil composition, fertilization, infection load, and irrigation regime. The pot trial also enables adding a healthy non-infected control group and design of the pots’ dispersion in complete randomization, avoiding cross-influence between the treatments. (2) The biological control was evaluated compared with the conventional chemical approach in a commercial cotton field. This is the first report of CRD bio-friendly treatments on such a scale in Israel. It is an initial essential step toward the future testing and application of integrated management interphase in a commercial field.

### 2.2 Fungal source and growth conditions

The phytopathogenic fungus *Macrophomina phaseolina* (isolate Mp-1) was obtained in 2017 from cotton plants affected by the fungus at Roni Cohen’s lab in the Newe Ya’ar Research Center, northern Israel. This isolate was identified through various methods, such as pathogenicity, physiology, colony morphology, microscopic characteristics, and molecular traits, as reported by Degani et al. (Degani et al., 2020; Degani and Gordani, 2022; Degani et al., 2023). All the fungi species were maintained in the dark on potato dextrose agar (PDA) medium at 28 ± 1°C under high humidity for 4–7 days. Per the manufacturer’s guidelines, the PDA medium was made by dissolving 39 g of PDA powder in 1 L of double-distilled water. For sub-culturing the fungus, a 6-mm (in diameter) colony agar disc taken from the margins of the culture was transferred to a fresh PDA-containing Petri dish, according to Degani et al. (2023). The Petri dishes were then incubated in the dark at 28°C using the MaxQ™ 6000 Incubated/Refrigerated Stackable Shakers (Thermo Fisher Scientific, Waltham, MA, USA). For submerged cultures, five fungal discs were grown in 150 mL of potato dextrose broth (PDB) in a 250-mL Erlenmeyer flask. The PDB medium was prepared by dissolving 24 g of PDB powder in 1 L of distilled water. The flasks were sealed with a breathable stopper and shaken at 150 rpm for 7–10 days under the above conditions. For extracting the fungal-secreted products, the growth liquid medium was filtered using a Buchner funnel with double Whatman paper, centrifuged at 6,000 rpm for 20 min, and filtered through a 0.4-micron membrane for sterilization. The *Trichoderma* species selected for this study were previously proven efficient in controlling CRD in sprouts trial (Degani et al., 2023). These species are *T. Longibrachiatum* (T7407 and T7507), *T. asperellum* (P1), *Trichoderma* sp. O.Y. 14707, and *Trichoderma* sp. O.Y. 7107 (**Table 1**).

**Table 1:** List of *Trichoderma* spp. isolates used in this study.

Species	Designation	Origin	Reference	Tested in the farm (pots)	Tested in the field
*Trichoderma* sp. O.Y. 14707	T14707	*Psammocinia* sp. ^1^	(Gal-Hemed et al., 2011; Degani et al., 2023)	Yes	No
*Trichoderma* sp. O.Y. 7107	T7107	*Psammocinia* sp. ^1^	(Gal-Hemed et al., 2011; Degani et al., 2023)	No	Yes
*Trichoderma longibrachiatum*	T7507	*Psammocinia* sp. ^1^	(Gal-Hemed et al., 2011; Degani et al., 2023)	Yes	No
*Trichoderma longibrachiatum*	T7407	*Psammocinia* sp. ^1^	(Gal-Hemed et al., 2011; Degani and Dor, 2021; Degani et al., 2023)	Yes	Yes
*Trichoderma asperellum*	P1	*Zea mays*, Prelude cv.	(Degani et al., 2021; Degani and Gordani, 2022; Degani et al., 2023)	Yes	Yes

^1^ Mediterranean sponge *Psammocinia* sp.

### 2.3 Whole-season pot assay under semi-field conditions

#### 2.3.1 Cotton growth conditions

The study was conducted at the North R&D plantation farm in Hula Valley, Upper Galilee, northern Israel (33°09′08.2″N 35°37′21.6″E) with the Pima V-70 cotton cultivar (ELS Pima-type, *G. barbadense*, from Israel Seeds). The objective was to test the effectiveness of four *Trichoderma* species (listed in **Table 1**), either alone or in combination with AS (Amistar S.C.; Syngenta, Basel, Switzerland, supplied by Adama Makhteshim, Airport City, Israel), compared to pathogen free mock control pots and unprotected control plants in soil enriched with *M. phaseolina*. The experiment treatments were replicated six to eight times, each repetition consisting of a pot containing five plants. The flowerpots were filled with 10 L of heavy local soil from the farm, which had no known history of CRD. If any infestation occurred, it was expected to be minimal. The ground was mixed with 30% coarse perlite to ensure sufficient aeration. The plants were irrigated with a computerized drip lines system every 2 days, adjusted to moderate soil wetness (usually 1.33 L per pot/day). Throughout the season, fertilizers and pest control treatments were applied to prevent diseases other than *M. phaseolina* infection.

#### 2.3.2 *Macrophomina phaseolina* infection and protective treatments

Inoculation was conducted by enriching the soil’s upper part with 150 g (per pot) of sterilized wheat kernels infected with *M. phaseolina* [prepared as described by Degani et al. (2023) and Gordani et al. (2023) 11 days prior to sowing. Another soil infection was performed upon emergence (1 week after sowing) for all treatments' pots by incorporating three *M. phaseolina* culture discs (diameter of 6 mm) into the soil. The protective treatments were based on *Trichoderma* species (**Table 1**) separately and combined with AS, whereas the control group included infected plants with only chemical protection. Two other control groups had plants infected by the pathogen without shielding (diseased plants) and healthy non-infected plants without biological or chemical intervention.

The bio-shieling was performed using a combined treatment of three *Trichoderma* fungi—T7407, T7507, and P1 (Mix)—in comparison with each fungus separately and the control fungus T14707, which has weak antagonistic activity (Degani et al., 2023). The bio-protection was applied in two steps. (1) Submerging the seeds in the *Trichoderma* spp. growth fluid (prepared as described in Section 2.2.) for 10 min. (2) Adding six mycelial discs from the *Trichoderma* colony of each isolate (or a mixture of two discs from each of the three isolates) to each seed during sowing. Chemical protection was performed by adding AS (commercial formula) with the daily 2 L of irrigation per pot, at 19.5 µL per pot, three times during the season (25, 53, and 68 days from sowing). The above-ground peeking percentages in each treatment were checked after a week. Symptoms, growth indices, and fungus DNA’s presence in each plant’s root using quantitative Real-Time PCR (qPCR) were measured on days 69 and 173 past sowing. The capsules’ development was estimated on day 129, and the crops were evaluated at the end of the season (day 173).

#### 2.3.3 Important dates and meteorological data

The semi-field bio-chemo control experiment took place during the spring and summer of 2022. **Table S1** provides specific dates of significance. The temperature and humidity conditions observed throughout the growing season were typical and aligned closely with disease development, as reported by Degani et al. (2020). Detailed meteorological data can be found in **Table S2**.

### 2.4 Hulda commercial field experiment

The experiment was conducted in spring-summer 2022 in the kibbutz Hulda commercial field in the Shephelah region, Israel, at 31°49′52.2″N 34°52′21.9″E. The area has been infested with CRD in recent years. The experiment was conducted in randomly designed split plots with the Pima V-70 cotton cultivar. It includes six treatments and a non-protected control group. All groups were tested in two irrigation regimes: early and late water openings. Each experimental group and the control consist of 12 repeats (the precise samples’ number analyzed is indicated in each figure legend). There were 168 plots in total, covering an area of about 2.1 ha (**Figure S1A**).

Two treatments involved chemical sprinkling in the sowing strip with the seeding: AS (Mirador 250 SC, active ingredient of 250 g/L, Adama Makhteshim, Airport City, Israel) at 200 and 400 cc per 0.1 ha. In addition, there were four biological treatments: *T. Longibrachiatum* (T7407) or a mix of *Trichoderma* species (T7407, *T. asperellum* (P1), and *Trichoderma* sp. O.Y. 7107; **Table 1**) seed dressing, and two similar bio-control treatment with a sprinkling of the *Trichoderma* species secreted metabolites in the sowing strip with the seeding. The control group received no treatment. Each experimental plot included six rows and measured 20 m long and 5.80 m wide, with 0.96 m of row spacing (**Figure S1B**).

#### 2.4.1 Growth protocol and conditions

The seeding was carried out on 13 April 2022 with water sprinkling at 100 L per 0.1 ha added to each sowing strip to ensure uniform germination. The sowing depth was 5 cm, and the number of plants was 13.3 per meter. On the 13th day after sowing, an above-ground emergence evaluation was performed, revealing normal sprouting in most parts of the field. On day 42, a developmental assessment was conducted, indicating the presence of seven to eight nodes in the plants, with the first bud observed in nodes 6–7. On day 48, irrigation was initiated for the early watering plots, applying a dose of 40 mm/day. On day 63 (15 days after beginning the early watering), irrigation was started for the late watering plots with the same water dose. The irrigations were performed twice a week. Precise dates of importance are present in **Table S3**.

The total amount of irrigation was 569 mm with 18.5 units of nitrogen in the early water opening plots and 539 mm with 16.5 units of nitrogen in the late water opening plots. Penman evaporation estimation was 1,040 mm over the entire growing period (13 April to 11 October) and 541 mm during vigorous growth and crop accumulation (1 June to 31 August). The temperature and humidity conditions measured during the growth period were normal and suitable for CRD development (**Table S4**).

Fertilizers and treatments against various pests were carried out throughout the experiment to maintain a risk factor only from the *M. phaseolina* infection. Fourteen pest control treatments were conducted in the experimental plot in total. One of the treatments involved the application of AS (Mirador 250 SC) through a drip irrigation line placed between the rows. However, this treatment was found to have minimal impact on the CRD symptoms or the overall results of the experiment. This is attributed to the dispersion of the chemical through the irrigation extension, located at a distance from the plants (between the rows) and hindered its effective concentration within the plants.

#### 2.4.2 The biological protective treatments

The *Trichoderma* cultures were grown in a liquid PDB medium, as described in Section 2.2. The cultures were ground in a blender for 2 min to obtain a blend of spores and short mycelial segments. The fungus’ colony forming units (CFU) and growth medium suspension were used for the seed coating. Two 9-L tanks were set for T7407 and the species mixture (Mix: 3 L of each species, T7407, P1, and T7107; **Table 1**). To enrich the CFU and extrolite suspension, spores and mycelium fragments from 30 fungal colonies (grown on solid PDA substrate 9-mm-diameter plates, in the dark, at 28°C for 4–5 days) were added to each container. To the Mix container, spores and mycelium fragments harvested from 10 colonies of each of the three species were added. The pH of the coating suspension was 4.7 for the T7407 and 4.3 for the Mix. To each container, 22.5 ml of 5× diluted Tween 80 was added to facilitate the fungus mycelium attachment to the seeds.

The liquid (9 L) was mixed well and added to 15 kg of cotton seeds (which had not been chemically treated). Each seed weighs ca. 126 mg, so 30 kg contains approximately 2.4 × 10^5^ seeds. The *Trichoderma* suspension and the seeds were mixed manually, and, after 10 min, the seeds were separated from the liquid, spread on a mat, and left to dry for 22 h. The coated and dry seeds were packed in sacks and transferred to sowing in the field.

The seed coating preparation was accompanied by the following tests: (1) a test that verified that Tween 80 does not affect the germination percentage of the seeds; (2) placing selected coated seeds on PDA plates to confirm the viability of the fungi; (3) the seed dressing liquid runoff was checked to verify the vitality of the fungi in the process. This remaining fluid (after the seeds treatment) had low pH 1.6–1.9. Still, the *Trichoderma* species could be recovered from this liquid, so their vitality was not compromised during the short treatment (10 min).

Secreted metabolites were prepared and harvested as described in Section 2.2. This *Trichoderma* growth medium (4.5 L) was mixed with 395-L tap water, and 300 L of this fluid was added by sparkling to each sowing row with the seeding. Each *Trichoderma* growth fluid that was tested and the *Trichoderma*-based coated seeds were from the same species.

#### 2.4.3 Symptoms and yield estimation

The disease impact was estimated at day 113 by counting the number of dead plants in the two middle rows in two to three repeats. On day 176, root samples were taken (one representative plant for each repetition, a total of 11–12 plants per treatment). These samples (also utilized for DNA extraction) were used for cross- and longitudinal-section photography. The yield assessment was done at the season-ending (day 181) by harvesting the two middle rows in each repetition using a cotton-picking machine.

Remote sensing aerial imaging of the cotton field was performed on day 64, using high-resolution visible-range RGB (red, green, and blue) and thermal imaging to evaluate the impact of the two water regimes on the plants’ growth. This assessment of changes in plant foliage temperature was conducted during mid-day (12:00–14:00). To carry out this evaluation, a DJI Mavic 2 Enterprise Dual quadcopter uncrewed aerial vehicle (UAV) (DJI, Shenzhen, Guangdong, China) was utilized as the flying platform for both RGB and thermal imaging. The built-in RGB camera featured a 4,056 × 3,040 pixels 4K resolution CMOS sensor and a 24-mm (35 mm eq.) lens, providing an 85° field of view (FOV) within a three-axis stabilized gimbal (https://www.dji.com/mavic-2-enterprise/specs). The built-in thermal camera had a recording size of 160 × 120 pixels, a lens with a horizontal FOV of 57°, and a spectral band ranging from 8 µm to 14 µm. The Pix4Dcapture software (Pix4D, Prilly, Switzerland) was utilized to create the flight program and serve as an automatic pilot for the UAV. The flight path was designed with a 90% front and side overlap to facilitate mosaicking. Subsequently, the images collected during each flight campaign were georeferenced and combined into a mosaic using Pix4Dmapper software developed by Pix4D.

### 2.5 Molecular real-time PCR diagnostic

#### 2.5.1 DNA extraction

The plants’ roots were washed thoroughly with running tap water and sliced into ca. 2-cm sections. The total weight of each repeat was adjusted to 0.7 g. For DNA extraction, the BioMed bag (universal extraction bags, Bioreba, Reinach, Switzerland) was filled with 4 ml of buffer cetyl hexadecyltrimethylammonium bromide solution and the root sample. The sample was homogenized for 5 min using an electric tissue homogenizer (Bioreba, Reinach, Switzerland). As previously described (Murray and Thompson, 1980) with slight modifications (Degani et al., 2019), the homogenized samples were treated for DNA purification. The DNA was suspended in 100 µL of high-performance liquid chromatography (HPLC)-grade pure water and kept at −20°C until use in the qPCR analysis.

#### 2.5.2 qPCR technique

The ABI-7900HT model, Applied Biosystems device with 384 well plates (Foster City, CA, USA), and the previously described method for the qPCR procedure (Degani et al., 2020; Degani et al., 2022; Dimant and Degani, 2023) were used here. The method is based on a conventional qPCR protocol, designed to identify messenger RNA (Complementary DNA) but has been fine-tuned to detect *M. phaseolina* DNA efficiently. Each sample well contained a total reaction volume of 5 µL, including 2 µL of sample DNA extract, 2.5 µL of iTaq™ Universal SYBR^®^ Green Supermix (Bio-Rad Laboratories Ltd., Rishon Le Zion, Israel), and 0.25 µL of both forward and reverse primers (10 µM each). The qPCR program consisted of a precycle activation stage (1 min at 95°C), followed by 40 cycles of denaturation (15 s at 95°C), annealing and extension (30 s at 60°C), and a subsequent melting curve analysis. Each roots’ sample was analyzed and tested four times to ensure the consistency of those technical repeats. The Mpk primers (sequences in **Table S5**) were employed explicitly for *M. phaseolina* detection. The COX reference gene (present in both the plants and the fungi that inoculate them) was used to normalize the DNA quantity (Weller et al., 2000). It is also used to determine the relative quantification of the target *M. phaseolina* fungal DNA using the ΔCt model (Livak and Schmittgen, 2001; Yuan et al., 2006). This “housekeeping” gene encodes the last enzyme in the eukaryotic mitochondria’s respiratory electron transport chain. The COX F/R primer set is detailed in **Table S5**. It was assumed that the efficiency was consistent across all samples.

### 2.6 Statistical analysis

The semi-field (open-enclosure) pots and the commercial field plots were scattered in a fully randomized design. The experiments were analyzed using the same statistical method performed by the JMP software, version 15 (SAS Institute Inc., Cary, NC, USA). The result differences were defined using the one-way analysis of variance (ANOVA) and a Student’s t-test *post-hoc* (without multiple tests correction, *p* < 0.05).

## 3 Results

In the present study, a biological control method based on the selected *Trichoderma* species was applied for the first time in Israel under field conditions throughout a complete growing season to control the CRD in cotton caused by the fungus *Macrophomina phaseolina*. An environmentally friendly bio-pesticides were tested as an alternative to a conventional chemical azoxystrobin (AS)-based pesticide. In addition, the efficacy of the biological agents was evaluated in combination with the chemical treatment at a lower dose to enhance the bio-friendly treatment stability and effectiveness under the open air changing environmental conditions. Four *Trichoderma* species were selected for this work based on their high performance in former studies (Degani et al., 2023). *T. Longibrachiatum* (T7407 and T7507), *T. asperellum* (P1), *Trichoderma* sp. O.Y. 14707, and *Trichoderma* sp. O.Y. 7107 (**Table 1**). The AS minimal inhibition concentration was formerly set and verified that it could be combined well with the bio-agents (Gordani et al., 2023). The evaluation was made in parallel in a semi-field (open-enclosure) pot trial and a commercial field over an entire season (173 and 181 days, respectively).

### 3.1 Whole-season pot assay under semi-field conditions

**Figure 1:**
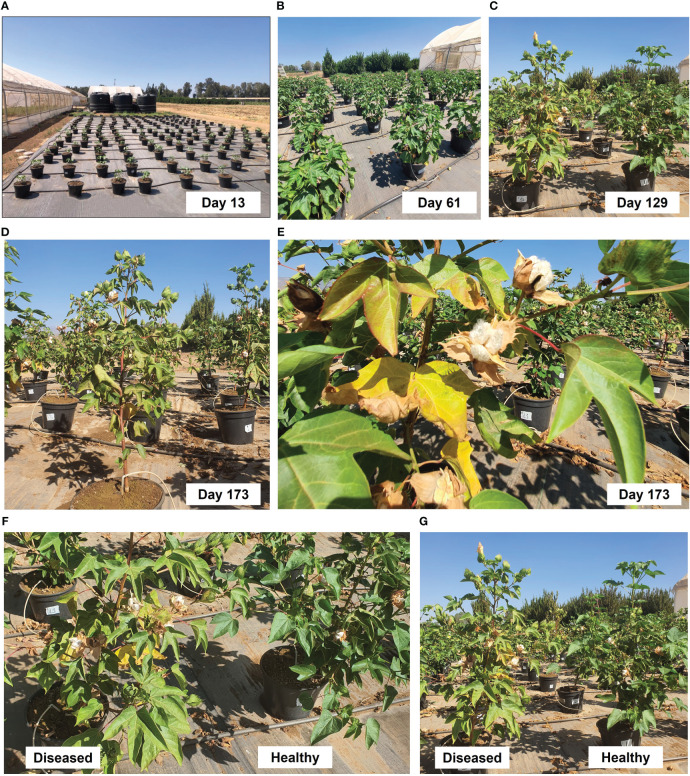
The cotton semi-field (open-enclosure) full-season pot experiment design and charcoal rot disease (CRD) outbreak from day 129 onward. The experiment took place at the North R&D plantation farm in Hula Valley, Upper Galilee, located in northern Israel. The cotton cultivar used was Pima V-70. Four *Trichoderma* species (listed in **Table 1**) were tested individually or in combination with chemical protection, compared with pathogen-free plants and infected control plants that did not receive any CRD prevention treatment. The soil used in the trial was enriched with *M. phaseolina*. Azoxystrobin (AS) (Amistar S.C., 19.5 µL per pot) was applied to the pots with the irrigation in three intervals: 25, 53, and 68 days after seeding. **(A)** The trial design and the sprouting phase. **(B)** Healthy growth 61 days post-seeding. **(C)** Dehydration symptoms in the non-protected plants’ group started to be observed from day 129 onward with increasing severity **(D, E)**. At the harvest (day 173), the impact of biological and chemical protection treatments was clearly visible in the overall vitality of the plants and the presence of dry leaves **(F)**.

This open-enclosure full-season pot trial tested integrated control interphase against CRD in Israel. This interphase is based on AS-*Trichoderma* combined treatment compared with each treatment alone. This semi-field study aimed at mimicking as closely as possible the field conditions. At the same time, such growth allows us to add healthy (non-infected) plans control and to maintain tight control of the infection load, the soil composition, the watering regime, and the isolation of each treatment (while scattering the treatments in a fully randomized design; **Figures 1A, B**). As hoped, the artificial soil inoculation evoked intense disease outbreaks in the unprotected control plants (**Figures 1C–F**). Indeed, the control plants suffer from 45% dehydration, as will elaborate below.

**Figure 2:**
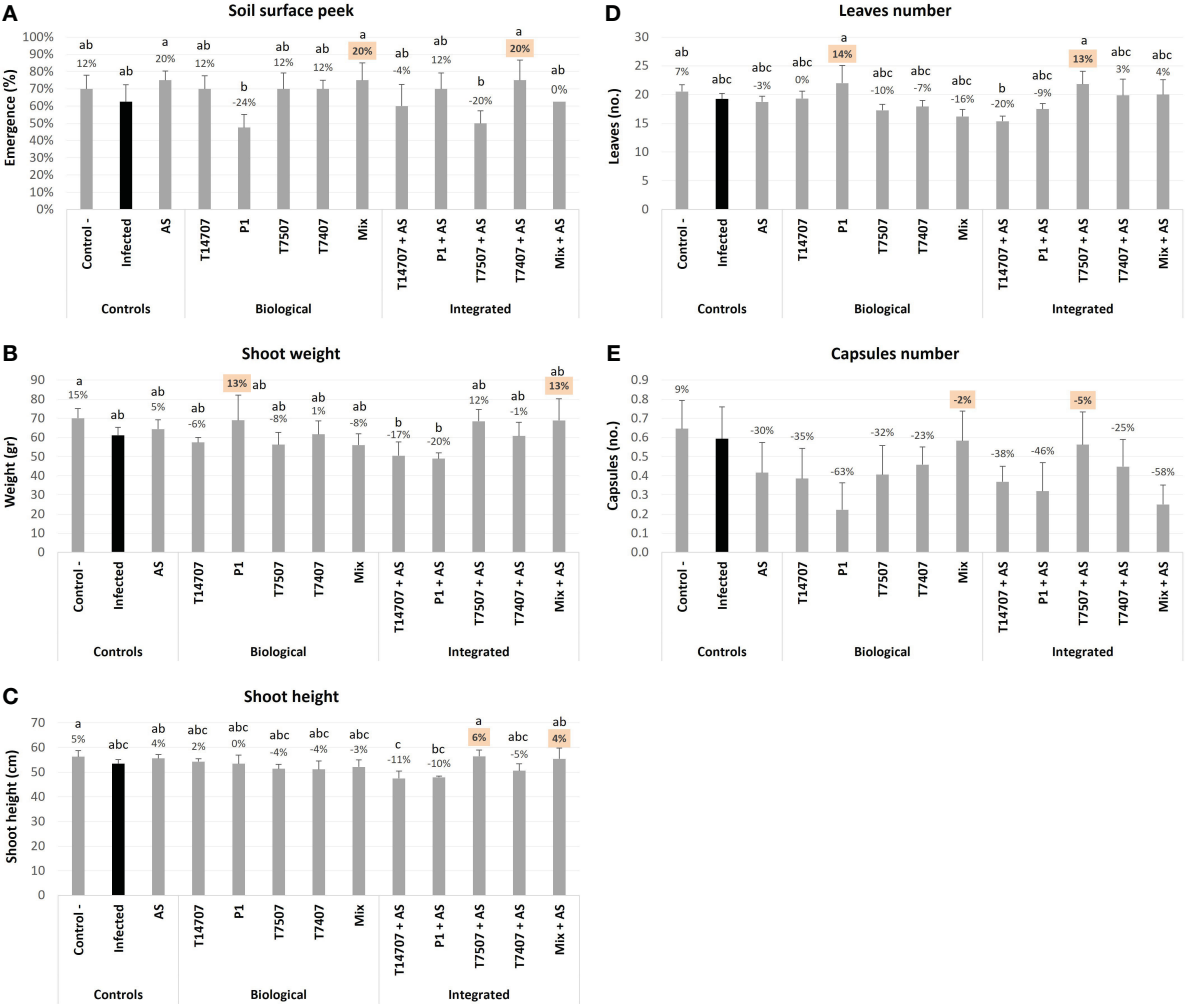
The semi-field trial above soil surface peek (day 7) and mid-season sampling (day 69) growth evaluation. The experiment is described in **Figure 1**. The mock control pots were kept pathogen-free. The infected plants’ group was CRD unshielded. The estimation included above-ground emergence percentages **(A)**, the shoot’s fresh weight **(B)** and height **(C)**, plants’ phenological development (total number of leaves) **(D)**, and the number of cotton capsules **(E)**. Means represent six to eight pots per treatment (16–28 plants per treatment); deviation bars indicate standard error. The percentages’ differences from the untreated-infected control (highlighted in black) are above the chart’s bars. The highest two parameters in each measure are highlighted in orange. If statistical significance exists, different letters (a–c) represent the ANOVA test score (p < 0.05).

At the above soil surface peek evaluation, made 7 days after sowing (DAS) (**Figure 2A**), some treatments had an inhibition impact. Specifically, *T. asperellum* (P1) and *T. Longibrachiatum* (T7507) + AS caused delayed emergence (24% and 20% inhibition compared with the non-protected control, without statistical significance). Still, this delay did not affect the plants in the later stages of growth and the evaluation of the effectiveness of the treatments. The growth indices on day 69 of the experiment (**Figures 2B–E**) indicate that the biological treatment with P1 resulted in a 13%–14% improvement in growth (shoot weight and leaves number). Applying the chemical treatment alone (AS at a low dose) showed either no effect or had an insignificant positive impact. However, when AS was added with the biological treatments, it enhanced by 21%–23% the effectiveness of *T. longibrachiatum* (T7507) alone or in a mixture with this species's additional isolate (T7407) and *T. asperellum*. On the other hand, it adversely affected the efficiency of P1. Day 69’s other growth measures, plant height and capsules’ number, were less informative regarding the treatments’ impact.

**Figure 3:**
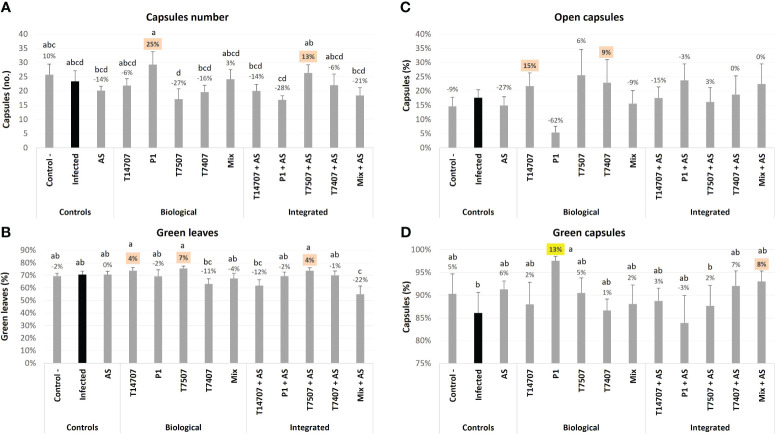
The semi-field trial growth parameters at day 129 from sowing. The experiment is described in **Figure 1**. The mock control pots were kept pathogen-free. The estimation included the total number of cotton capsules (**A**; mean capsules per plant was 21.9), the proportion of green leaves **(B)**, the open cotton capsules percentage (**(C**; mean open capsules per plant was 3.8), and green capsules proportion **(D)**. Each value is a mean of seven to eight repetitions (pots containing one plant per treatment). Error bars indicate standard error. The percentages’ differences from the untreated-infected control (emphasized in black) are above the chart’s bars. Highlighted in yellow is a statistically markedly difference and in orange are the most noticeable results (without significance). Different letters (a–d) represent an ANOVA test statistically meaningful discrepancy (*p* < 0.05).

On day 129, the number of cotton plants’ capsules (at different ripening stages) in the P1 and T7507 + AS treatment pots improved by 13%–25% (compared with the non-protected control; **Figure 3**). As expected, most of the capsules at this stage were green, especially in the P1 pots, which had significantly more green capsules (13%) than the untreated-infected group. Consequently, the number of open capsules in the P1 group was relatively low.

**Figure 4:**
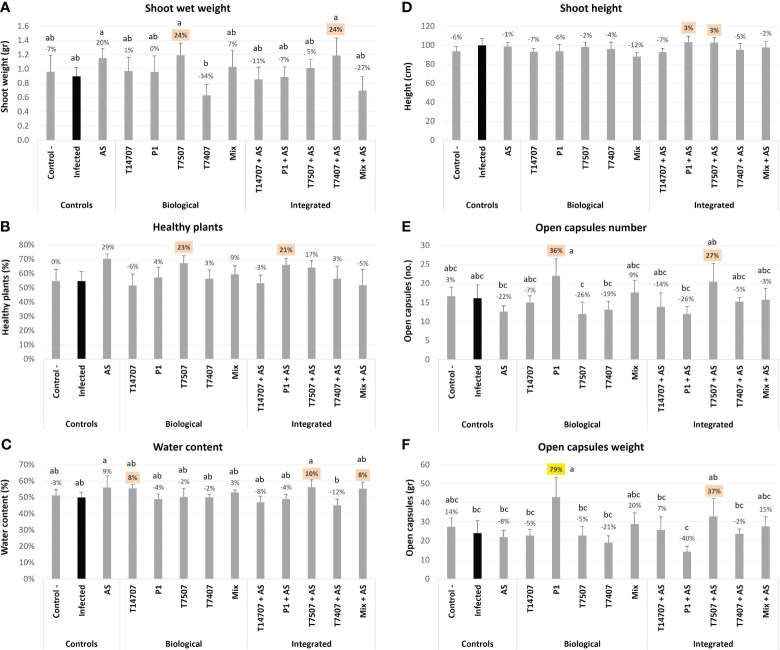
The semi-field trial growth parameters at the harvest (day 173 from sowing). The experiment is described in **Figure 1**. The mock control pots were kept pathogen-free. The estimation included the shoot’s fresh weight **(A)**, healthy plants percentages **(B)**, the plants’ water content **(C)**, shoot height **(D)**, open cotton capsules number **(E)**, and weight **(F)**. Each value is a mean of six to eight repetitions (plants per treatment). Error bars indicate standard error. The percentages’ differences from the untreated-infected control (highlighted in black) are above the chart’s bars. Emphasized in yellow is a statistically marked dissimilarity and in orange are the most noticeable results (without significance). Different letters (a–c) represent an ANOVA test observable variation (*p* < 0.05).

By harvest time (day 173; **Figure 4**), *T. longibrachiatum* (T7507) treatment improved the shoot weight and the overall health of the plants by 23%–24% compared with the unshielded control. Adding the AS chemical further enhanced the effectiveness of *T. longibrachiatum* (T7407, 58% higher shoot weight) and *T. asperellum* (P1, 18% more healthy plants) interventions compared with the same treatment without AS. Moreover, AS alone demonstrated its efficacy, resulting in a notable 20%–29% improvement in these indices. The various protective applications minorly affected the plants’ water content (maintaining a steady 50%–60% value) and shoot height. In contrast, the cotton capsules’ number and weight remain the highest in the P1 and T7507 + AS treatment pots, with 36% and 27%, and 78% and 37% improvement over the non-treated control (respectively). At this stage, 30%–40% of the capsules were open, and the two successful treatments (P1 and T7507 + AS) were also the highest in this measure (*p* < 0.05 in the P1 treatment). AS alone did not improve capsule yield, yet it negatively impacted the P1 improvement regarding this measure (62% and 119% less open capsules and capsules weight compared with the same treatment without AS).

**Figure 5:**
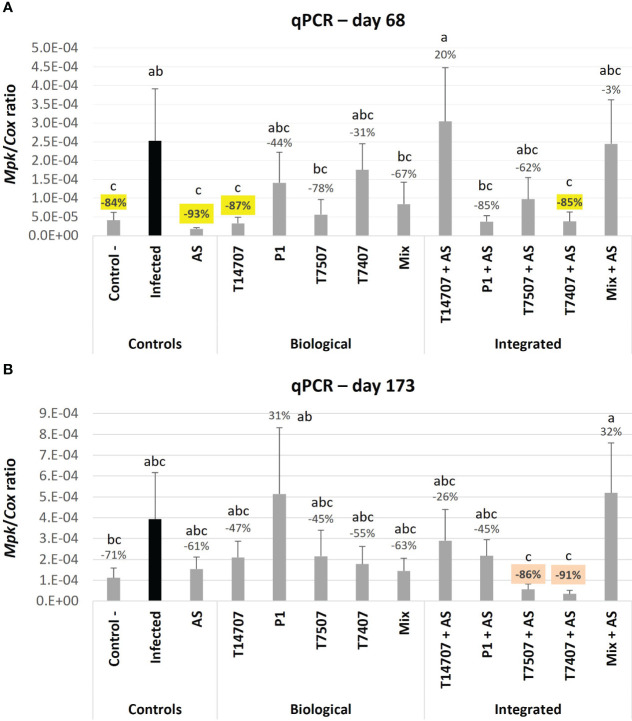
Real-time PCR–based monitoring of *M. phaseolina*’ DNA inside the plants’ roots. This evaluation was made in the semi-field trial at mid-season (**A**; day 68) and harvest (**B**; day 173). The mock control pots were kept pathogen-free. The relative amount of *M. phaseolina* DNA (*Mpk*) normalized to the cytochrome C oxidase DNA (*Cox*) in the plants’ roots is presented in the Y-axis. Each value is a mean of six to eight repetitions (plants per treatment). Error bars indicate standard error. All other parameters as in **Figure 4**.

Monitoring the pathogen’s DNA within the plant roots enables a sensitive assessment of treatment effectiveness (**Figure 5**). On day 68, the majority of the biological and combined treatments demonstrated high efficiency in suppressing the pathogen (31%–93%). The AS sole treatment excels in this measure, followed by the T14707 biological treatment (93% and 87% pathogen suppression). Combining AS with either T7407 or P1 remarkably improved their efficiency (from 31% and 44% to 85%).

On day 173 of growth, the efficiency decreased to 45%–63% (or more in a few cases) across all treatments, except for the combined treatments T7407 + AS and T7507 + AS, which maintained 86%–91% high efficacy. The effectiveness of the treatments, as indicated by the growth and health indices on different sampling days, is ranked and presented in **Table 2**. Among the treatments, P1 and T7507 + AS exhibited the highest effectiveness. The addition of AS improved the efficacy of the T7507 treatment but hindered the effectiveness of P1.

**Table 2:** Comparative evaluation of the treatments’ success in the semi-field pot trial^a^.

Rank	Day 68	Day 129	Day 173	Total
**1**	T7507 + AS	P1	P1	P1
**2**	P1	T7507 + AS	T7507 + AS	T7507 + AS
**3**	Control	T14707	Mix	Control
**4**	Mix + AS	Control	Control	T14707
**5**	AS	T7407 +AS	T14707	T7407 + AS
**6**	T7407 + AS	Mix	Mix + AS	Mix
**7**	T14707	AS	T7407 + AS	AS
**8**	T7407	T7507	T14707 + AS	Mix + AS
**9**	T7507	T7407	AS	T7507
**10**	Mix	P1 + AS	T7507	T7407
**11**	P1 + AS	T14707 + AS	T7407	T14707 + AS
**12**	T14707 + AS	Mix + AS	P1 + AS	P1 + AS

a The plants’ growth and health results in all experiments were analyzed by calculating the differences in percentages of each index in each treatment to the control—untreated-infected cotton plants. The total column orders the experimental groups according to the average of all score measures. The rank columns categorize the degree of the treatment’s effectiveness (giving all assays’ weighted scores). In green are the best protective applications (including the non-infected mock control). In blue are the mid-successful pesticides, and in orange are the least efficient ones.

### 3.2 Hulda commercial field experiment

A commercial field scale experiment evaluated the *Trichoderma*-based protection against the CRD pathogen for the first time in Israel. At this stage, the bio-shielding and the more traditional AS chemical application were tested separately, laying the foundation for developing and implementing the integrated bio-chemo strategy on a field scale in follow-up studies. To this end, the biocontrol agents were applied in seed coating, an economical and easily feasible method. Moreover, bio-coating has the advantage over chemo-coating because, unlike the last, the *Trichoderma* spp. that inhabit the seeds keep developing after the sowing and provide continuous protection throughout the plant life (**Figure S2**).

**Figure 6:**
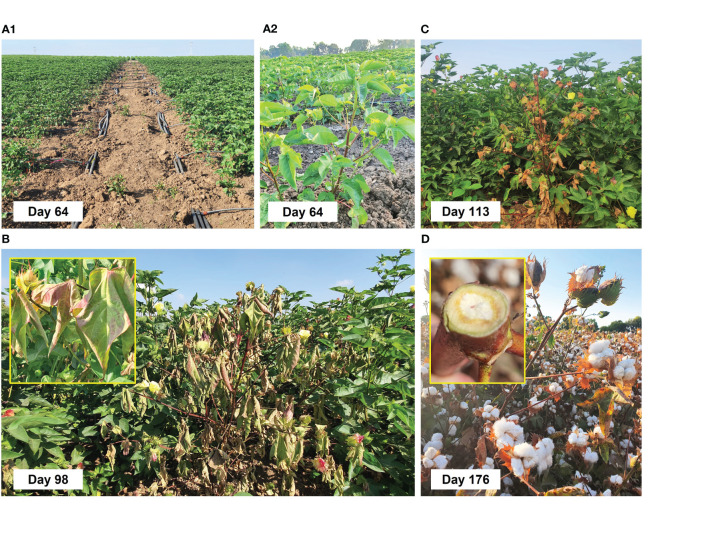
Field irrigation **(A1, A2)** and charcoal rot disease symptoms **(B–D)** on day 64 after sowing (DAS) onward. The Pima V-70 cultivar was evaluated, an extra-long staple (ELS) from Israel Seeds. The trial tested six chemical and biological control treatments separately to reduce cotton charcoal rot disease (CRD). A control group was left without protection. Two driplines irrigation regimes, early (48 DAS) and late (63 DAS) water openings, were applied to all groups. Each treatment group and the control group were replicated 12 times. The treatments included AS (Mirador 250 SC, active ingredient of 250 g/L, Adama Makhteshim, Airport City, Israel, at 200 and 400 cc per 0.1 ha) chemical sprinkling in the sowing strip with the seeding, four biological treatments: *T. Longibrachiatum* (T7407) or a mix of *Trichoderma* species [T7407, *T. asperellum* (P1), and *Trichoderma* sp. O.Y. 7107; **Table 1**] seed dressing, and two similar bio-control treatment with a sprinkling of the *Trichoderma* species secreted metabolites in the sowing strip with the seeding. The cotton plants impacted by *Macrophomina phaseolina* had drying stems and leaves (**B**, insert) inset and vascular bundle color alternation to brown (**D**, insert).

The field experiment comprised six treatments and a control group (**Figure S1**). The plots were split into early watering (48 DAS) and late watering (63 DAS) openings (**Figure 6A**). The latest poses drought challenges to the plants, possibly contributing to enhanced CRD development (Cohen et al., 2022). On the 70th day of growth, one flower per meter was observed in the late water opening plots. The plants measured 47 cm in height, with six nodes above the first yellow flower, and exhibited a growth rate of 1.5 cm per day. In contrast, the early water-opening plots plants, assessed on day 77, showed advanced growth. The plants in this water regime reached a height of 75 cm, with nine nodes above the first yellow flower, and displayed a growth rate of 2.3 cm per day. Another development assessment was performed on day 113 of growth, where about 10% of the capsules were open.

Already on day 64, the disease symptoms started to appear. These dehydration symptoms spread to all the plant’s above-ground parts, eventually killing the host (**Figures 6B–D**). However, although the disease caused patches of wilting plants in the field, other plants thrived undisturbedly. One common indication of CRD is the discoloration of the plant’s vascular tissue, which occurs due to the toxins reacting with a substance produced by the plant in response, likely gossypol (Cohen et al., 2022). Gossypol, a sesquiterpene phytoalexin present in cotton, is one of the most significant compounds that protect the plant against harmful invaders (Li et al., 2019). Such vascular tissue discoloration to brown was documented in diseased plants at the season-ending (**Figure 6D**, inset).

**Figure 7:**
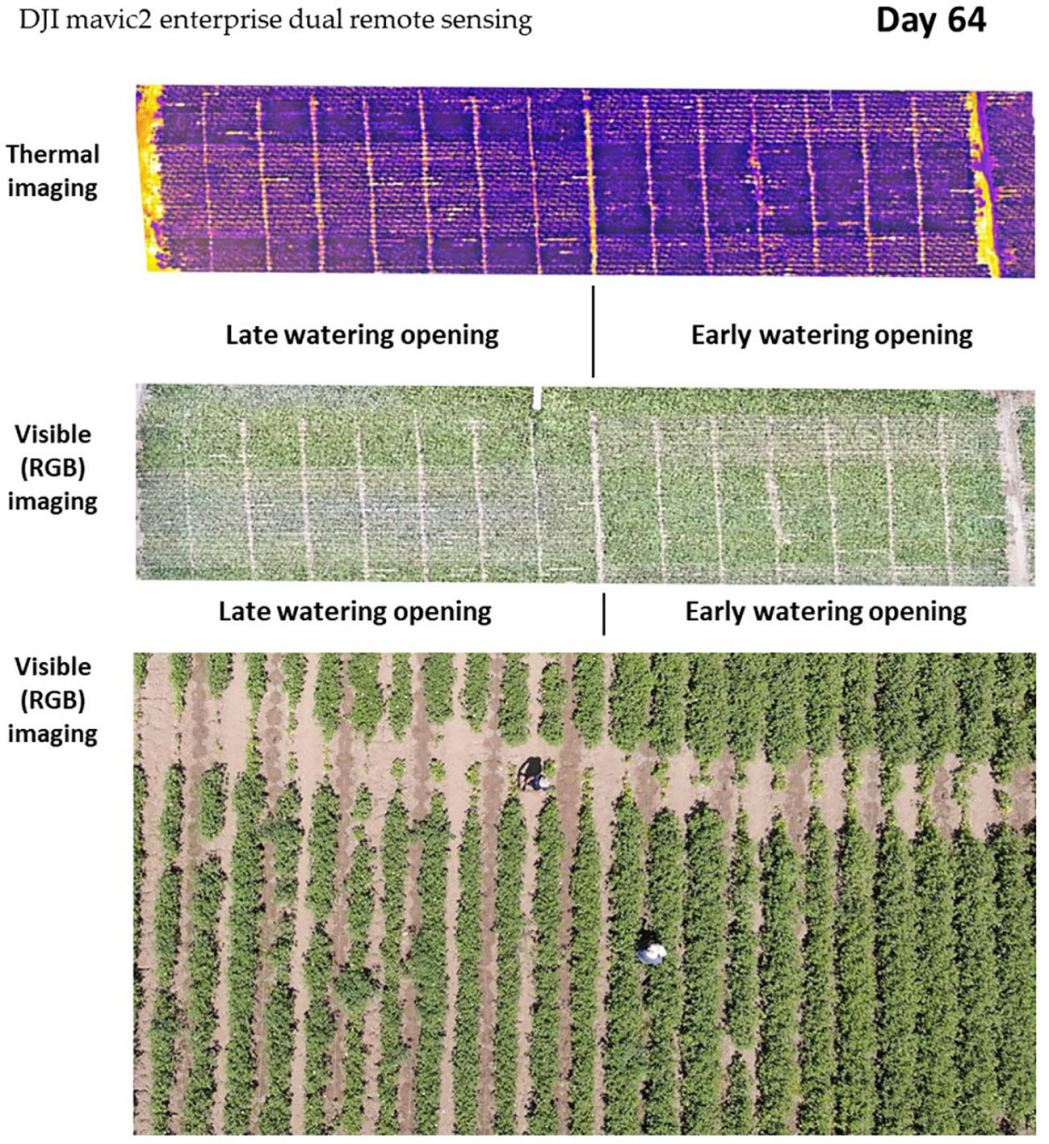
Aerial imaging of the cotton growth disparity under the two water regimes, early watering opening (right side, 48 DAS) and late watering opening (left side, 63 DAS). Images were taken 64 days after seeding, using remote aerial sensing [thermal imaging and high-resolution visible-range RGB (red, green, and blue)]. The thermal imaging shows the lower foliar temperature of the early watering plots (evident by the darker purple color). The visible RGB channel represents the more vigorous vegetation (darker green) and enhanced development compared with the late watering plots (see the bottom closeup picture).

The early irrigation initiation on the 48th day of growth, approximately 3 weeks before flowering, led to accelerated plant development compared with the plots that received late irrigation opening (15 days later). The disparity in growth can be observed through thermal and RGB drone imaging (**Figure 7**).

**Figure 8:**
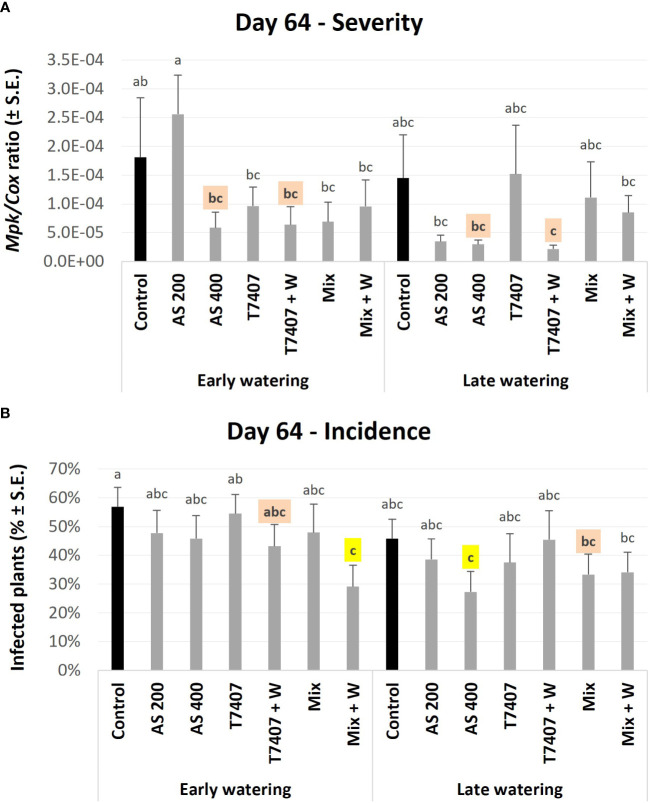
First (day 64) field trial real-time PCR–based monitoring of *M. phaseolina*’ DNA inside the plants’ roots. The experiment is described in **Figure 6**. The chemical treatment was Azoxystrobin in two dosages, 200 and 400 cc per 0.1 ha (AS 200 and AS 400). The *Trichoderma*-based biological intervention included: *T. longibrachiatum* (T7407) and Mix [mixture of T7407, *T. asperellum* (P1), and *Trichoderma* sp. O.Y. 7107; **Table 1**]. The bio-treatments were applied as seed coating alone or with the sprinkling of their secreted metabolites in the sowing strip (+ W). The control plots were kept pesticide (chemical or biological) free. **(A)** The relative amount of *M. phaseolina* DNA (*Mpk*) normalized to the cytochrome C oxidase DNA (*Cox*). **(B)** The disease incidence (percentages of infected plants). Each value is a mean of 10–12 repetitions (plants per treatment). Error bars indicate standard error. The untreated-infected control is emphasized in black. Highlighted in yellow is a statistically meaningful disparity and in orange are the most noticeable results (without significance). Different letters (a–c) represent an ANOVA test substantial discrepancy (*p* < 0.05).

qPCR-based molecular monitoring of the pathogen presence within the plants’ roots proved sensitive and informative. Already at growth day 64, some treatment protective impact was clearly measured. Such successful treatment was the AS (both dosages, 200 and 400 cc per 0.1 ha) with 68%–84% pathogen reduction, although the AS 200 was ineffective at the early watering plots. In addition, the *T. longibrachiatum* with the sprinkling of its extrolites in the sowing strip (T7407 + W) marked 75%–98% pathogen restriction. Those results reflected the infection severity (*M. phaseolina* DNA; **Figure 8A**) and were slightly different from the disease incidence (number of infected plants) index (**Figure 8B**). The highest performance in CRD incidences reduction (*p* < 0.05) was achieved in the mixture of *Trichoderma* species [T7407, *T. asperellum* (P1), and *Trichoderma* sp. O.Y. 7107], with the sprinkling of their secreted metabolites (Mix + W, in the early watering plots, 49%) and the AS 400 (in the late watering plots, 52%). The second best CRD control was reached by T7407 + W (24%) and Mix (41%) in those two water regimes (respectively, without statistically meaningful disparity).

**Figure 9:**
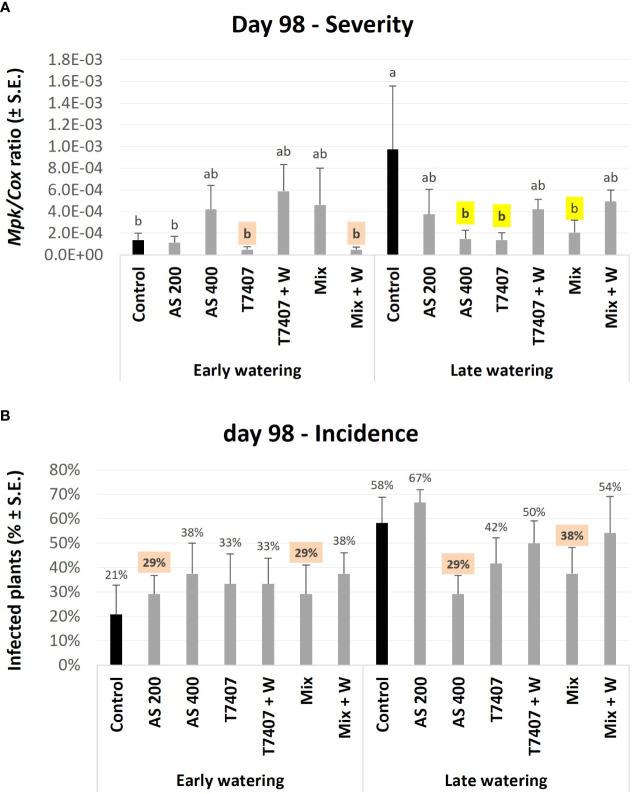
Second (day 98) field trial real-time PCR–based monitoring of *M. phaseolina*’ DNA inside the plants’ roots. **(A)** The relative amount of *M. phaseolina* DNA (*Mpk*) normalized to the cytochrome C oxidase DNA (*Cox*). **(B)** The disease incidence (percentages of infected plants). The experiment is described in **Figure 6**. Each value is a mean of five to six repetitions (plants per treatment). Error bars indicate standard error. All other parameters as in **Figure 8**. Different letters (a–b) represent an ANOVA test substantial discrepancy (p < 0.05).

The second molecular evaluation was conducted at 98 DAS. At this stage, some diseased plants scattered in the field already showed severe wilting (**Figure 6B**). In the unprotected plots under the early irrigation opening regime, the pathogen DNA levels were 86% (*p* < 0.05) lower than those in the late watering opening regime (**Figure 9A**). This result is expected because irrigation timing is crucial for CRD development (Cohen et al., 2022). Because of that, the protective intervention impact was less pronounced in the early watering opening plots (*p* > 0.05). Still, the T7407 and the Mix + W in the early watering plots marked 65% *M. phaseolina* DNA reduction compared with the control. At the late watering plots, the differences from the much higher control values were statistically clear (p < 0.05). Here, the AS 400, T7407, and Mix treatments marked a high 79%–86% pathogen inhibition. The other treatments were also effective (49%–62% fungal repression). Similar tendencies were measured in the disease prevalence (**Figure 9B**) with some variation (no statistically significant was reached).

**Figure 10:**
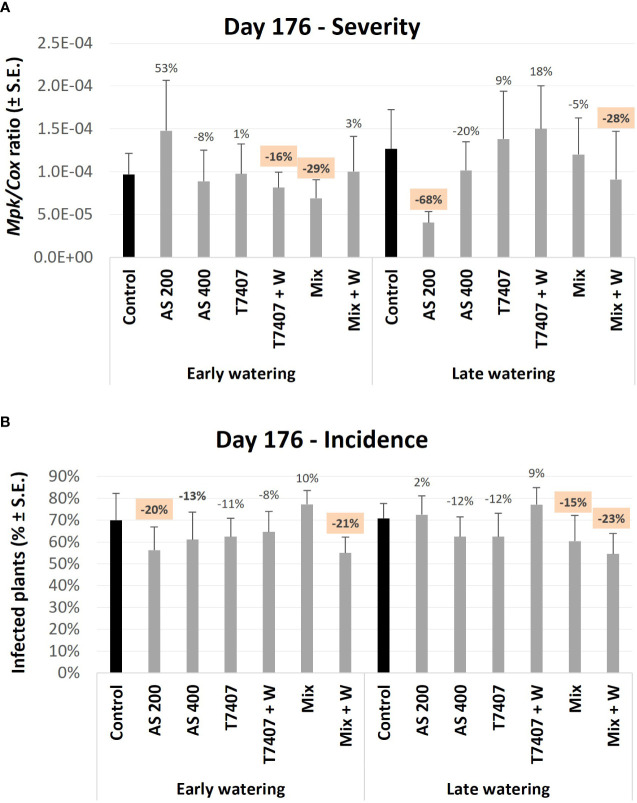
Third, end season (day 176) field trial Real-time PCR–based monitoring of *M. phaseolina*’ DNA inside the plants’ roots. **(A)** The relative amount of *M. phaseolina* DNA (*Mpk*) normalized to the cytochrome C oxidase DNA (*Cox*). **(B)** The disease incidence (percentages of infected plants). The experiment is described in **Figure 6**. Each value is a mean of 9–12 repetitions (plants per treatment). Error bars indicate standard error. All other parameters as in **Figure 8**.

At the near-harvest sampling (150 DAS), the molecular picture of day 98 was generally kept, with the AS and Mix treatments leading as the most successful (**Figure 10A**). Still, at this growth phase, the differences from the control were smaller and non-statistically significant. Among all, the T7407 + W and Mix (in the early watering) and AS 200 and Mix + W (in the late watering) were the most influencing (16% and 29%, and 68% and 28%, pathogen DNA reduction, respectively). Molecular tracking of the disease incidences (**Figure 10B**) also supported these trends (with AS 200 and Mix + W generally as the most impactable).

The molecular monitoring results variations among the many treatments along the season are complex, and elucidating the main conclusion from this analysis is challenging. Thus, we ranked each treatment according to its efficiency and summed the scores of all experiment groups in each water regime and in total (**Table 3**). This analysis rated the AS 400 and *Trichoderma* Mix (with or without adding their secreted products to the sowing strip) as most beneficial in CRD prevention. It also ordered the *T. longibrachiatum* (T7407) as the least effective among the procedures tested. The AS 400 was best in reducing the pathogen infection (severity) and number of infected samples (incidence), but reducing the compound concentration to half drastically impaired this result.

**Table 3:** Comparative evaluation of the real-time PCR analysis in the field trial^a^.

Rank	Early watering		Late watering		Total		
	Severity	Incidence	Severity	Incidence	Severity	Incidence	Total
**1**	AS 400	AS 200	AS 400	AS 400	AS 400	AS 400	**AS 400**
**2**	Mix	Mix + W	AS 200	Mix	Mix + W	Mix + W	**Mix**
**3**	T7407 + W	AS 400	Mix	Mix + W	T7407	Mix	**Mix + W**
**4**	T7407	T7407 + W	Mix + W	T7407	T7407 + W	AS 200	**AS 200**
**5**	Mix + W	Mix	T7407	AS 200	Mix	T7407	**T7407**
**6**	AS 200	T7407	T7407 + W	T7407 + W	AS 200	T7407 + W	**T7407 + W**

a The real-time PCR results were analyzed by calculating the differences in percentages of each treatment in each sampling day to the control—untreated naturally infected cotton plants. The treatments were ranked according to efficiency (from the lowest infection severity or infection incidences to the highest). The sum of all ranks (scores) for each treatment was calculated, and the treatments were presented from the most effective (at the top) to the least (at the bottom). The total column is the treatment ranked according to the sum of all score measures. In green are the best protective applications. In blue are the mid-successful pesticides, and in orange are the least efficient ones.

**Figure 11:**
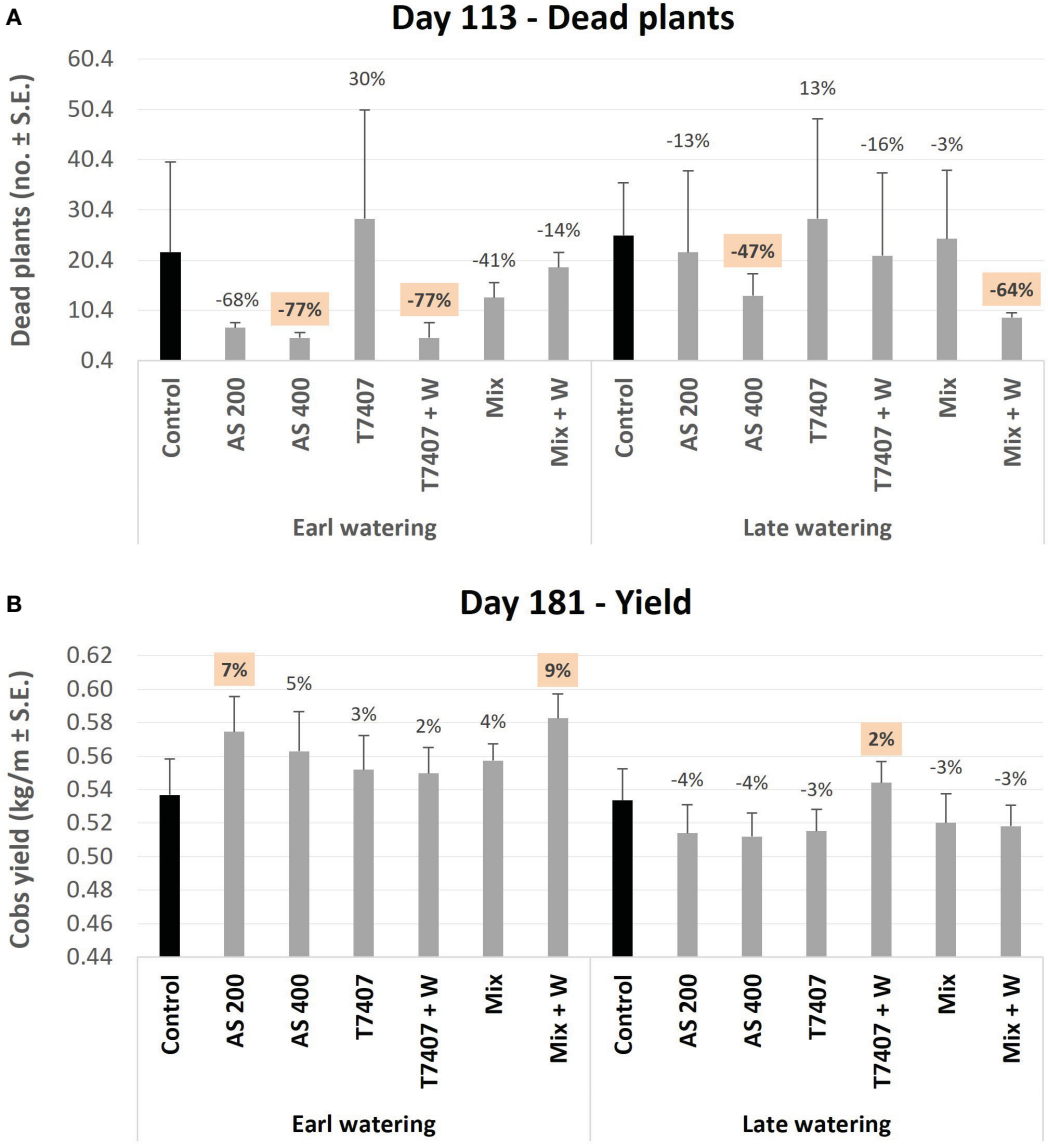
The disease symptoms (dead plants; **A**) and yield **(B)** assessments. The disease’s impact was assessed on day 113, where all wilt plants were counted in the two central rows of two to three repetitions. The yield evaluation occurred at the season-ending on day 181, as the two middle rows in each repeat were harvested using a cotton-picking machine. Each value in the yield evaluation is a mean of 11–12 repetitions (plants per treatment). Error bars indicate standard error.

Two more conclusions can be withdrawn from this interpretation (**Table 3**). First, although the *T. longibrachiatum* (T7407) treatments received a low rank in the total evaluation, they reached third place in reducing infection severity. Indeed, this treatment (with the secreted metabolite addon, T7407 + W) was the most effective in reducing the pathogen severity at day 64 (**Figure 8**). In addition, the T7407 may significantly contribute when combined with other *Trichoderma* species (as in the Mix treatments here). Still, the Mix treatment’s success can result from the other *Trichoderma* species in this complex. Specifically, the role of *T. asperellum* (P1) may also be pivotal, as shown in the semi-field pot experiment (**Table 2**). Second, it appears that adding the *Trichoderma*-secreted products to the sowing strip did not provide a valuable contribution here. However, in the late water opening plots at harvest, the *Trichoderma*’s extrolites sprinkling with the seeding did give better outcomes in the Mix + W compared with Mix alone (reduced infection severity by 23% and CRD prevalence reduction by 8%; **Figure 10**). Both these aspects mattered regarding symptoms’ estimation and crop evaluation (**Figure 11**).

Concerning the sensitive molecular tracking results, it is essential to emphasize that the intricate nature of interactions (that manifests in variability in treatments’ efficacy) underscores the necessity for a cautious interpretation, especially when using these findings in practical situations.

**Figure 12:**
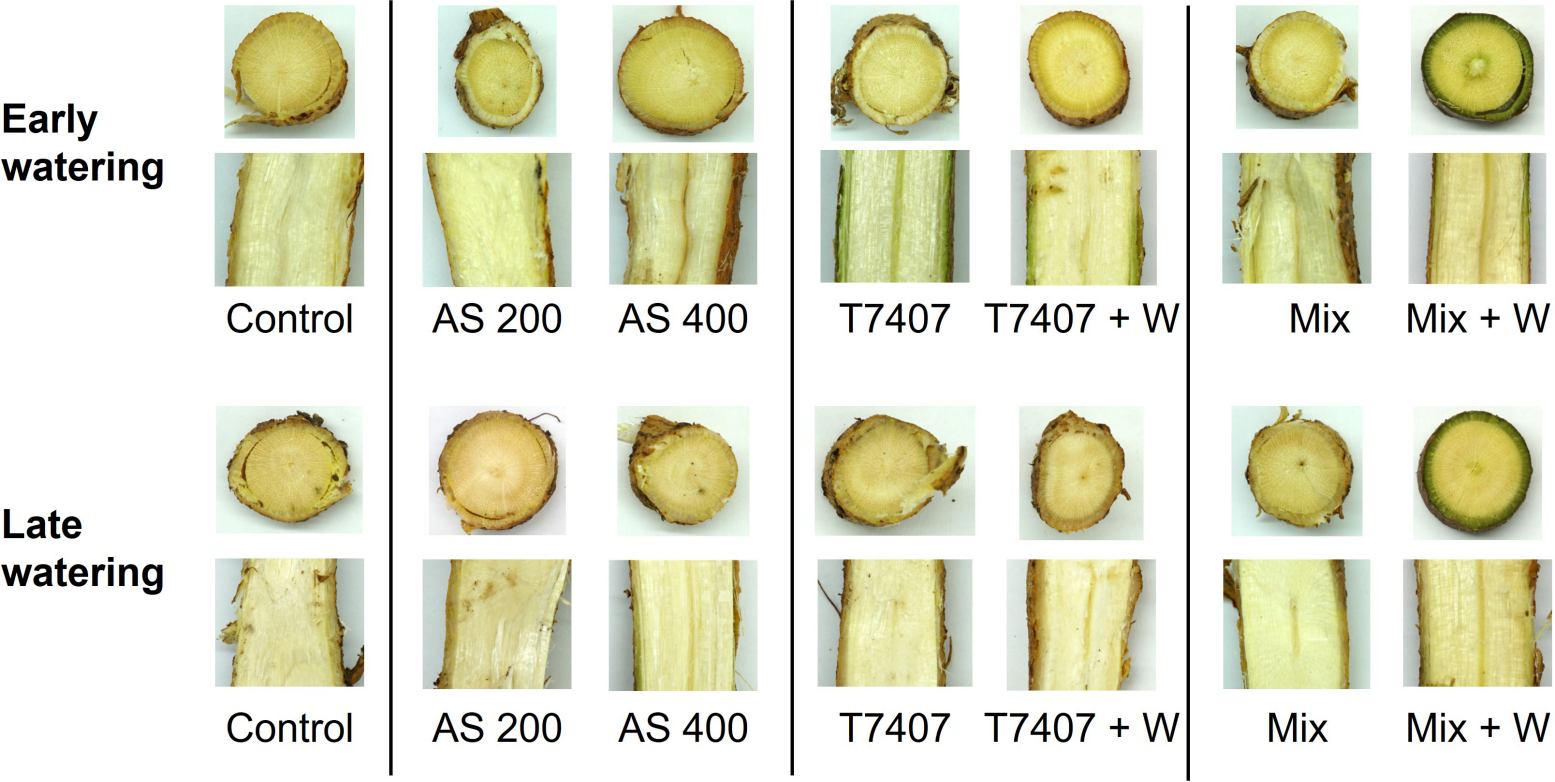
Plants’ roots’ cross and longitudinal sections photos at day 176 from sowing. The roots’ samples were taken from arbitrarily selected mature plants at the capsules’ ripening phase. In this assessment, more vivid roots have greener color (particularly in the phloem tissue).

Finally, the disease symptoms (dead plants, **Figure 11A**, 113 DAS, and stalk symptoms, **Figure 12**, 176 DAS) and yield (**Figure 11B**, 181 DAS) were assessed. There is a clear difference between the early and the late watering opening, with generally better health and yield parameters in early irrigated plots. These evaluations pointed out the *Trichoderma* species mixture, the *T. longibrachiatum* (both with their secreted metabolites added to the seeding strip, T7407 + W and Mix + W), and the chemical treatments (200 and 400 cc per 0.1 ha) as the most effective. Both biological treatments excelled in those parameters, but their best performance was subjected to the irrigation initiation timing and the parameter assessed. The differences were more visible in the dehydrated plants’ evaluation, with 77% (AS 400 and T7407 + W) and 47%–64% (AS 400 and Mix + W) more vivid plants in the early and late irrigated plots (respectively). The *Trichoderm*a extracellular matrix addon effectively improved the plants’ health in the treatments T7407 (107% and 29% in the early and late watering) and Mix (61% in the late watering; **Figure 11A**). The successful treatments’ yield data (AS 200 and Mix + W) indicates 7%–9% more crops than the control. This achievement was evident in the early watering plots, but the delay in water opening almost entirely abolished this crop’s improvement (**Figure 11B**).

Inspecting representative plants’ roots’ cross and longitudinal sections (**Figure 12**) provided another support for the treatments’ benefit. The roots’ samples taken from mature plants at the capsules’ ripening phase (day 176) indicate overall more vivid roots in the early watering plots. Moreover, the bio-shielding using the *Trichoderma* species mixture and their extrolites (Mix + W) kept the roots greener (specifically the phloem tissue) than the other treatments (and control). The T7407-based treatments also had some (weaker) positive impact here.

## 4 Discussion

The CRD, caused by the necrotrophic fungus *Macrophomina phaseolina*, is a major worry to Israeli cotton growers (Degani et al., 2022). Moreover, the disease restrains’ effort crosses borders, and it is one of the worldwide scientific focus, particularly because the pathogen has a vast host range that makes it a serious threat to many horticulture and crop plants (Marquez et al., 2021). Although control approaches to restrict the pathogen’s negative influence are constantly developed and tested, so far, our toolkit to handle the condition is limited (Iqbal et al., 2014; Iqbal and Mukhtar, 2020b; Marquez et al., 2021). The current work was focused on expanding this toolkit using a *Trichoderma*-based biocontrol strategy, alone or in combination with low-dosage of azoxystrobin (AS), to provide the bio-agent stabilization and strength in the open-enclosure unstable conditions (Karthikeyn, 2022; Degani et al., 2023). The integrated approach was already assessed in various plants against specific phytoparasitic fungi [reviewed by Ons et al. (2020)]. Such a method is beneficial because it reduces the chemical treatments’ environmental footprints and health risks. It is also essential to prevent fungicides’ fungal resistance and may be effective against other soil fungal phytopathogens (Aly et al., 2007; Bastakoti et al., 2017; Marquez et al., 2021).

The result presented here uncovered the complexity of the CRD-integrated bio-chemo control approach application. On the one hand, it can significantly enhance the bio-shielding impact, as resulted in the *T. longibrachiatum*–AS (T7507 + AS) combination. On the overhand, it may drastically disrupt the protective features of other species, as happened in the *T. asperellum*–AS (P1 + AS) treatment. Such an effect may be the outcome of the preparation toxicity toward some *Trichoderma* species (Ons et al., 2020). Hence, a careful examination of each such combined treatment is needed to verify its safety for the *Trichoderma* species, which will affect the overall effectiveness of the bio-chemo shielding. Still, the AS toxicity is probably not the reason for the low P1 performance in growth indexes. This is because *T. asperellum* was previously proven resistant to AS (Gordani et al., 2023). To support this, the molecular tracking results (**Figure 5**) showed that adding AS improved P1’s ability to suppress *M. phaseolina.* Thus, other factors yet to be discovered probably cause (P1 + AS) treatment growth promotion failure.

Along with the integrated control innovation presented here, the work results of both the semi-field pot trial and the commercial field assay demonstrated the high efficiency of the *Trichoderma* species in reducing CRD. This encouraging result agrees with other scientific works [see, for example, Iqbal and Mukhtar (2020a)]. The *Trichoderma* species mixture (with or without their extrolites addon to the sowing strip, Mix + W) applications excelled similarly to the conventional chemical treatment (AS at a high dosage) in terms of growth promotion, yield, disease symptoms elimination, and pathogen’s roots colonization (tracked by qPCR). It is essential to clarify that the AS high dosage tested here (400 cc per 0.1 ha) is above the acceptable dosages commonly used in commercial fields. Thus, the use of such a dosage is probably not realistic. Reducing the dosage by half drastically compromised its effectiveness, and, compared with the low dosage, the bio-treatments were superior in defending the cotton crops from CRD.

Thus, what are the next steps in establishing the results and applying this knowledge for farmers’ and markets’ benefit? First, the full potential of the integrated biological-chemical approach presented here in pots should be put to trial on a commercial field scale. Second, the *Trichoderma* species seed enrichment method can be optimized and improved. In addition, new species of *Trichoderma* should be screened for CRD protection potential, as done before (Iqbal and Mukhtar, 2020a). Last, the AS dosages and application method should be set and optimized to ensure minimal use and maximum impact. Similarly, other effective chemical compounds (Bashir et al., 2017; Iqbal and Mukhtar, 2020b) should be tested for the integrated control interphase to expand our options to restrict *M. phaseolina* damages. Studying the influence of the control methods presented here on other cotton soil diseases and other crops is also an intriguing and valuable future perspective.

An important aspect that gained attention in this work is the effect of the water regime on CRD outbursts and damages. In Israel and other semiarid regions, cotton is grown for six months, seeding in early spring when the soil is still damp from winter rains and harvesting in the fall. Throughout the summer, irrigation is used from the beginning of flowering until harvest. The roots of cotton plants are vulnerable to pathogen entry, typically through cracks in the phelloderm caused by soil expansion and cracking during hot, dry weather (Sarejanni and Cortzas, 1936) (**Figure S3**). The regions where the soil has dried out and formed fissures experience elevated soil temperatures and increased drought stresses. As a result, this harms the plants' roots and most probably facilitates the infiltration and establishment of *M. phaseolina* (Cohen et al., 2022).

Thus, how does the irrigation timing affect this cascade of events? Carefully planned irrigation may be a crucial factor here. Common drip irrigation that places the irrigation line between the rows is aimed to minimize the investment in irrigation equipment but exposes some roots to *M. phaseolina* infection. To support this assumption, implementing a dual-piping system for irrigation (a dripline for each row) to mitigate drought stress, lower soil temperature, and minimize root damage resulted in a 40% decrease in CRD severity, irrespective of the fungicide treatment applied (Cohen et al., 2022).

The CRD typically manifests its initial symptoms during early summer. These symptoms primarily involve leaf chlorosis, probably resulting from the pathogen toxins produced as it invades the roots (Chaudhary et al., 2023). These toxins are then transported to the plant’s above-ground structures. Subsequently, despite being watered, the affected plant may display signs of dehydration (Cohen et al., 2022). Early opening of water may reduce the soil drought and temperature stresses, the roots injury, and thus the fungus colonization of the plants at the beginning of the season. Subsequently, such optimal watering will affect the advanced development stages and the crops.

Indeed, in the field trial presented here, the yields were higher in the early watering plots. This growth promotion is not only due to improved water supply. At harvest, all control treatments in these plots better prevent CRD symptoms compared with the same procedures in the late watering plots. To support that, the pathogen molecular tracking done 98 and 176 days from sowing reveals a drastic increase of *M. phaseolina* infection in the late watering untreated control plots (7- and 1.3-fold).

The work results align with previous reports that examined AS against the CRD agent, *M. phaseolina* (Cohen et al., 2022). Treatments with AS in the sowing band at the beginning of growth may reduce the early infection of the fungus in the roots and, thus, reduce the disease without harming the crop. The possibility of using biological agents instead of chemical treatments or combining them to deal with the disease is an important strategy that should be explored more to uncover its full potential. Bio-control agents such as *Trichoderma* sp., *Bacillus subtilis*, and *Pseudomonas* sp. applied as seed treatments, soil applications, or foliar sprays have proven effective in combating pathogens and enhancing crop growth (Karthikeyn, 2022). Still, it was shown that a single isolate of an antagonist can yield significant effectiveness against one isolate of *M. phaseolina* while showing minimal impact on other isolates of the same fungus (Aly et al., 2007). Consequently, assessing the antagonist’s isolates against as many pathogen isolates as possible is crucial. This approach enhances the likelihood of identifying antagonist isolates that are effective against multiple variants of *M. phaseolina*.

In addition, implementing cultural practices like managing water stress, practicing crop rotation, and adopting inter-cropping techniques has shown effectiveness in reducing inoculum load and preventing infection by *M. phaseolina* (Iqbal et al., 2014; Karthikeyn, 2022). Finally, modifying the soil’s micro-environment by enriching it with inorganic salts, lime, gypsum, compost, and other additions can make it unfavorable for pathogens and reduce inoculum.

With the ongoing progression of global warming, various regions experience an increasingly favorable environment for *M. phaseolina*. Consequently, this will likely result in more severe disease outbreaks and the expansion of its natural range. Therefore, there is an immediate need to identify and improve effective control strategies to protect the most vulnerable agronomic and horticultural crops.

## 5 Conclusions

Israel cotton production is threatened by the CRD, caused by the fungus *Macrophomina phaseolina*. Currently, no adequate control interphase has been established and implemented in the country. *Trichoderma* spp. are considered promising environmentally friendly fungi for biocontrol purposes and promoting the growth of various crop plants, such as cotton. This study explored an integrated pest management approach combining a *Trichoderma*-based biological treatment with an AS-based intervention to minimize the disease impact on growth factors, plant health, and crop yield. In a semi-field pot trial, the eco-friendly treatments based on *T. asperellum* (P1) and *T. longibrachiatum* (T507) alone protected the cotton plants throughout the full season. Adding AS at minimal concentration improved the T7507 impact and impaired P1 efficiency. On day 68, the real-time PCR monitoring of pathogen DNA in the plants’ roots indicated that most biological and combined treatments had significant efficacy in suppressing the pathogen, ranging from 62% to 93%. By harvest on day 173, this efficiency decreased to about 45%–63%. Nevertheless, the combined treatments, *T. longibrachiatum* (T7407 and T7507) + AS, maintained a high 86%–91% efficacy level. In a large-scale commercial field experiment, the highest yields were reached in seeds treatment with a blend of *Trichoderma* species (mix of P1, T7407, and *Trichoderma* sp. O.Y. 7107 isolate), parallel to their secreted metabolites addon during seeding. This Mix treatment outperforms the AS (at a high dosage of 4,000 cc/ha) irrigation with seeding. Still, both these treatments excelled in reducing *M. phaseolina* infection levels and disease prevalence. Finally, an improved irrigation timing proved valuable here, with overall better growth, yield, and health result compared with a late watering opening in the spring. The significance of these protocols is cross-border as they provide cotton growers around the world with an improved set of tools to minimize the damages of the CRD. Integrated disease management allows us to minimize hazardous chemicals and emphasizes eco-friendly approaches to managing *M. phaseolina* with reduced risk for pesticide resistance.

## Data availability statement

The original contributions presented in the study are included in the article/**Supplementary Material**. Further inquiries can be directed to the corresponding author.

## Author contributions

OD: Conceptualization, Data curation, Formal Analysis, Funding acquisition, Investigation, Methodology, Project administration, Resources, Supervision, Validation, Visualization, Writing – original draft, Writing – review & editing. AG: Conceptualization, Data curation, Formal Analysis, Investigation, Methodology, Validation, Visualization, Writing – review & editing. ED: Conceptualization, Data curation, Formal Analysis, Investigation, Methodology, Validation, Visualization, Writing – review & editing. AC: Conceptualization, Data curation, Methodology, Writing – review & editing. OR: Conceptualization, Data curation, Formal Analysis, Funding acquisition, Investigation, Methodology, Project administration, Resources, Supervision, Validation, Visualization, Writing – review & editing.
