# Supplementary material for: The cotton charcoal rot causal agent, Macrophomina phaseolina, biological and chemical control
Source: Front Plant Sci. 2023 Sep 19;14:1272335. doi: 10.3389/fpls.2023.1272335 (PMC10546428; doi:10.3389/fpls.2023.1272335)
Supplement: Supplementary file 1 [file DataSheet_1.docx]

**Table S1.** The semi-field pot experiment’s dates.

| **Date** | **Inoculation and above-ground sprouting assessment** | **Days from sowing** |
| --- | --- | --- |
| 19/07/2022 | 1^st^ inoculation (sterilized infected millet grains) | -11 |
| 26/05/2022 | Seeding | 0 |
| 02/08/2022 | 2^nd^ inoculation (3 discs/sprout) and soil surface peek evaluation | 7 |
|  | **Pesticide (azoxystrobin) treatments** |  |
| 17/08/2022 | Pesticide I | 25 |
| 01/09/2022 | Pesticide II (28 days from Pesticide I) | 53 |
| 15/09/2022 | Pesticide III (15 days from Pesticide II) | 68 |
|  | **Sampling and harvest** |  |
| 11/09/2022 | Mid-season sampling and thinning | 69 |
| 01/10/2022 | Capsules’ development estimation | 129 |
| 19/10/2022 | Harvest and final sampling | 173 |

**Table S2.** Meteorological data for the semi-field experiments ^1^.

| **Parameters** | **Value** |
| --- | --- |
| Dates | 26/05/2022–15/11/2022 |
| Temperature (°C) | 25.5 ± 5.9 |
| Humidity (%) | 62.2 ± 18.9 |
| Soil temp. top 5 cm (°C) | 29.2 ± 4.9 |
| Radiation (MJ/m^2^) | 21.02 |
| Precipitation (mm) | 24.3 |
| Evaporation (mm) | 1,016.6 |

^1^ Average data (± standard deviation) according to Israel Northern Research and Development, Hava 1 Meteorological Station.

**Table S3.** The Hulda commercial field experiment’s dates.

| **Date** | **Seeding and sprouting assessment** | **Days from sowing** |
| --- | --- | --- |
| 13/04/2022 | Seeding | 0 |
| 26/04/2022 | Soil surface peek evaluation | 13 |
|  | **Irrigation** |  |
| 31/05/2022 | Early watering opening | 48 |
| 15/06/2022 | Late watering opening (15 days from early watering opening) | 63 |
|  | **Sampling and harvest** |  |
| 25/05/2022 | Development assessment in all plots | 42 |
| 16/06/2022 | DNA sampling I and Remote sensing (visible and thermal imaging) | 64 |
| 22/06/2022 | Developmental assessment in the late watering plots | 70 |
| 29/06/2022 | Developmental assessment in the early watering plots | 77 |
| 20/07/2022 | DNA sampling II and symptoms evaluation | 98 |
| 04/08/2022 | Disease symptoms evaluation | 113 |
| 06/10/2022 | DNA sampling III | 176 |
| 11/10/2022 | Harvest and yield assessment | 181 |

**Table S4.** Meteorological data for the Hulda commercial field experiments ^1^.

| **Parameters** | **Value** |
| --- | --- |
| Dates | 13/04/2022–11/10/2022 |
| Temperature (°C) | 24.2 ± 3.1 |
| Humidity (%) | 69.0 ± 7.7 |
| Soil temp. top 5 cm (°C) | 24.8 ± 3.6 |
| Radiation (MJ/m^2^) | 24.9 ± 3.7 |
| Precipitation (mm) | 0.3 |
| Evaporation (mm) | 1,040 |

^1^ Average data (± standard deviation) according to meteorological station Hulda 24 of The Land Conservation Division, Israel Ministry of Agriculture.

**Table S5.** Primers for quantitative real-time PCR *Macrophomina phaseolina* detection.

| **Pairs** | **Primer** | **Sequence** | **Uses** | **Amplification** | **References** |
| --- | --- | --- | --- | --- | --- |
| **Pair 1** | MpKFI MpKRI | 5’-CCGCCAGAGGACTATCAAAC-3′  5’- CGTCCGAAGCGAGGTGTATT-3’ | Target gene | 300-400 bp *M. phaseolina* species-specific fragment | (Babu et al., 2007) |
| **Pair 2** | COX-F  COX-R | 5′-GTATGCCACGTCGCATTCCAGA-3′  5′-CAACTACGGATATATAAGRRCCRRAACTG-3′ | Control | Cytochrome c oxidase (COX) gene product | (Weller et al., 2000;LiHartung and Levy, 2006) |


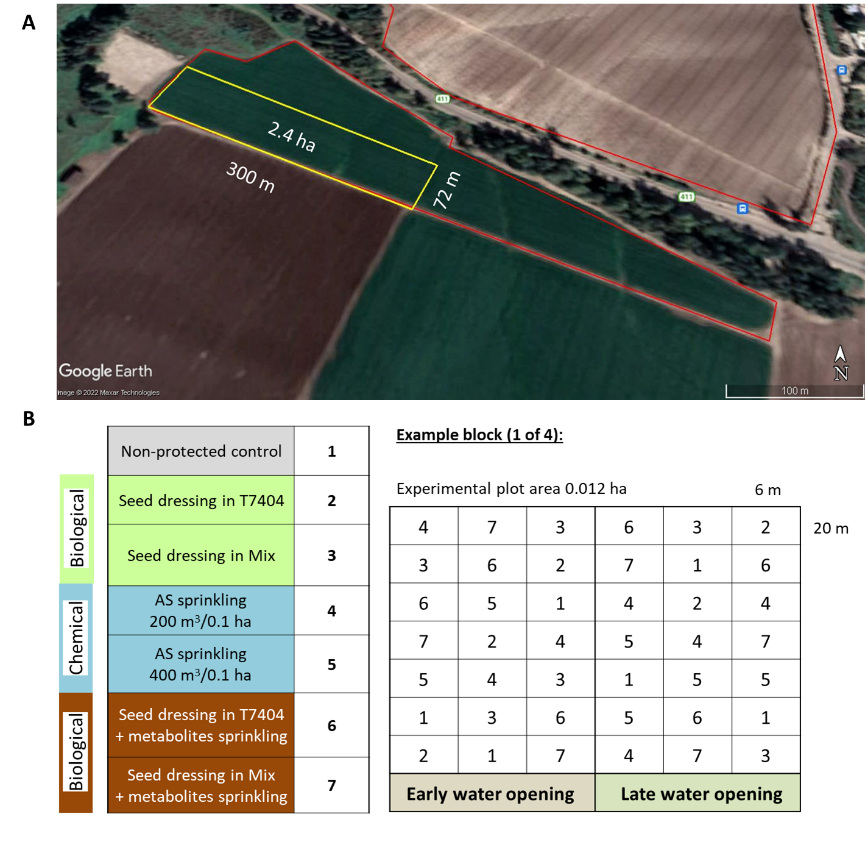


**Figure S1.** The Hulda commercial field’s experimental design. **A**. The study’s site is in the Shephelah region, Israel. The experiment was conducted at the kibbutz Hulda cotton field at 31°49’52.2“N 34°52’21.9“E. (highlighted in yellow), which was part of a large commercial field (designated in red). The Pima V-70 cultivar was evaluated, an extra-long staple (ELS) from Israel Seeds. **B**. The trial tested six chemical and biological control treatments separately to reduce cotton charcoal rot disease (CRD). A control group was left without protection. The study utilized a randomly designed split-plot approach. Two irrigation regimes, early and late water openings, were applied to all groups. Each treatment group and the control group were replicated 12 times. The treatments included azoxystrobin (250 g/l active ingredient, Adama Makhteshim, Airport City, Israel, at 200 and 400 ccs per 0.1 ha) chemical sprinkling in the sowing strip with the seeding, four biological treatments: *T. Longibrachiatum* (T7407) or a mix of *Trichoderma* species (T7407, *T. asperellum* (P1) and *Trichoderma* sp. O.Y. 7107, Table 1) seed dressing, and two similar bio-control treatment with a sprinkling of the *Trichoderma* species secreted metabolites in the sowing strip with the seeding.

**
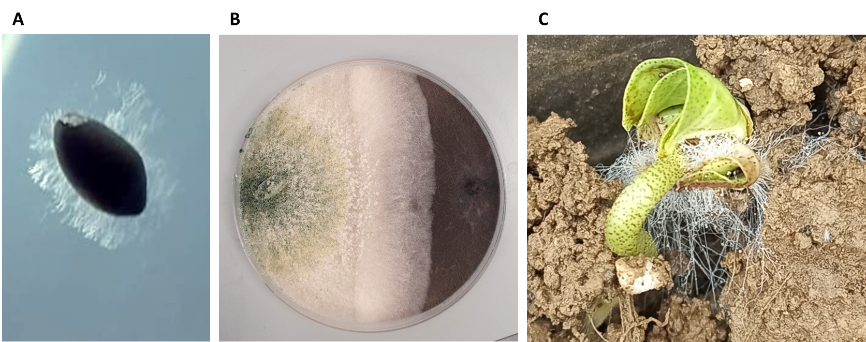
**

**Figure S2.** *Trichoderma* Mix seed coating. A *Trichoderma* species mixture (Mix) with equal amounts of three species, *T. asperellum*, *T. longibrachiatum*, and *Trichoderma* sp. O.Y. 7107 isolate (T7407, P1, T7107, Table 1) was utilized. Spores and mycelium fragments (along with their growth medium) were harvested from *Trichoderma* liquid and solid reach media cultures and used for the cotton seeds’ coating. The coated and dry seeds were tested on potato dextrose agar (PDA) growth medium plates to confirm the viability of the fungi (**A**). A plate confrontation (antagonism or mico-parasitism) against *M. phaseolina* seeded on the plate’s right pole ensured their biocontrol activity (**B**). Seven old seedlings were tracked to verify the *Trichoderma* app. success host colonization (**C**). All these tests prove the *Trichoderma* species’ success in seeds’ colonization, high bio-control potential, and proliferation ability during the sprouting phase.


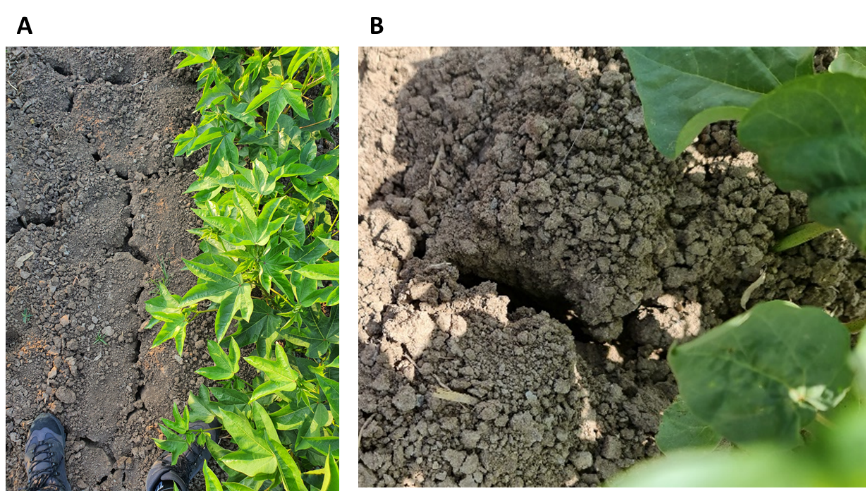


**Figure S3.** The soil dried-out regions with fissures elevate soil temperatures and drought stresses and, thus, harm the roots and may encourage infiltration by *Macrophomina phaseolina*. Photos were taken 64 days post-sowing. **A**. Overall view of the region between the rows. **B**. Close-up image of a crack in the soil.
